# Case report of arthritis caused by *Legionella anisa* and review of the literature

**DOI:** 10.1186/s12879-022-07475-3

**Published:** 2022-07-20

**Authors:** M. Roussotte, E. Massy

**Affiliations:** Department of Rheumatology, Hospices Civils de Lyon, Service de Rhumatologie Sud, Centre Hospitalier Lyon Sud, 165, Chemin du Grand Revoyet, 69310 Pierre Bénite, France

**Keywords:** Arthritis, *Legionella non pneumophila*, Case report

## Abstract

**Background:**

*Legionella* spp. is recognized as a common cause of community acquired pneumonia, with *Legionella pneumophila* serogroup 1 being the most prevalent. At least 70 species are described so far but few are identified in pathogenic conditions. Data on extrapulmonary infections are scarce.

**Case presentation:**

A 73-yar-old male with chronic lymphoid leukemia was hospitalized for an insidious wrist arthritis. Ultrasound of the wrist showed a carpal and radiocarpal fluid effusion with positive Doppler signal. While routine bacterial cultures remained sterile, 16S rRNA PCR identified *Legionella anisa*. Ciprofloxacin 500 mg twice a day for a period of six weeks improved arthritis with full recovery at the end of the treatment.

**Conclusion:**

*Legionella non pneumophila* are a rare cause of septic arthritis especially found in immunosuppressed patients and identification of species could help clinician to adapt antibiotherapy.

## Background

*Legionella* spp. is recognized as a common cause of community acquired pneumonia, with *Legionella pneumophila* serogroup 1 being the most prevalent. 70 species are described so far but few are identified in pathogenic conditions [1]. Data on extrapulmonary infections are scarce. Herein, we report a case of *Legionella anisa *monoarthritis.

## Case presentation

A 73-year-old male was hospitalized in the rheumatology department for an insidious inflammatory swelling of the right wrist. Symptoms began six weeks before with a localized swelling of the right index finger. He received NSAID followed by a week of pristinamycin without improvement. He reported no local trauma, respiratory symptoms or fever but occasional mild night sweats.

He had a medical history of chronic lymphoid leukemia (CLL), treated by chemotherapy five years before (bendamustine in association with rituximab). He was a former postman and had gardening and woodworking as hobbies.

On admission, the patient showed right wrist synovitis since two months without extra rheumatologic complaints. Blood tests showed leukocytosis (40.8 G/L) with lymphocyte predominance (32 G/L). Neutrophil count was also increased (7.6 G/L), as well as C-reactive protein (44 mg/L). Liver enzymes were within ranges. Immunological assays were negative, including rheumatoid factor and anti-CCP antibodies (except anti-nuclear antibodies at 1/160, without specificity). There was no hypogammaglobulinemia.

**Fig. 1:**
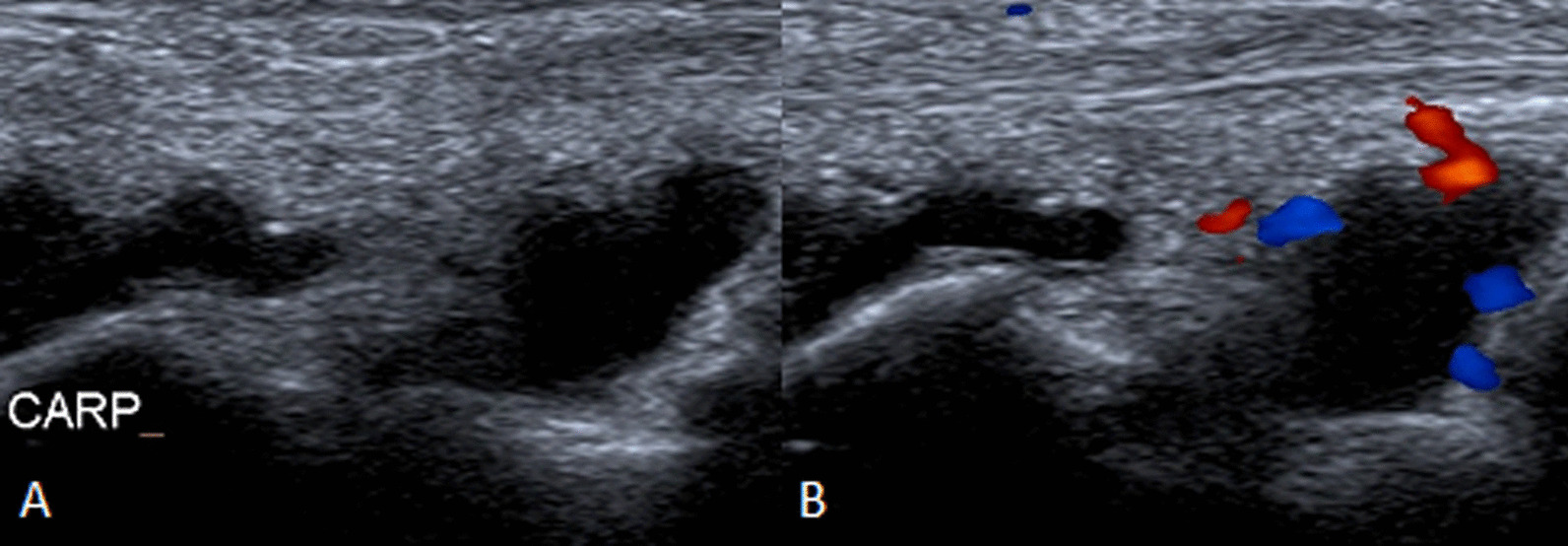
Ultrasound of the wrist (**A**, **B**), with positive Doppler signal (**B**)

Ultrasound of the wrist showed a carpal and radiocarpal fluid effusion with positive Doppler signal (Fig. 1). There was a palmar and dorsal subcutaneous infiltration as well, without collection. Fluid aspiration was hemorrhagic, with a white blood count of 36900/mm^3^. Neutrophil count was 43% and mono-histiocytes count was 49%. Routine bacterial cultures remained sterile but 16S ribosomal RNA (rRNA) polymerase chain reaction (PCR) identified *Legionella anisa* at day 4. The manufacturer of the equipment was Diagenode. Blood cultures were sterile.

Ciprofloxacin 500 mg twice a day for a period of six weeks improved arthritis with full recovery at the end of the treatment. Interestingly, C-reactive protein showed spontaneous normalization before any treatment.

The source of infection was presumably gardening. The patient had a well in his garden. Chest X-ray was normal. No environmental exploration was performed according to the national reference center guidelines.

## Discussion and conclusion

*Legionella* spp. are ubiquitous, aerobic, gram-negative rods naturally found in freshwater environments and are usually transmitted to humans in aerosols. They are regarded as fastidious bacteria as they do not grow on routine bacteriologic media. The clinical manifestations of *Legionella* infections are primarily respiratory (Legionnaires’ disease), but several extrapulmonary infections has been described. *Legionnella* spp has been implicated in arthritis, meningitis, sinusitis, endocarditis, pericarditis, myocarditis, pancreatitis, peritonitis and soft tissue infections [2].

While *L. pneumophila* is responsible to the vast majority of human infections, data on *L. anisa* pathogenicity are scarce. Despite being one of the most frequent species of *Legionella* in the environment, only eight articles reported infections secondary to *L. anisa* [3–10] (Table 1). In a French study, this strain was the most frequent non-pneumophila species in the environment (13.8%), but only accounted for 0.8% of the clinical isolates [11]. It has been responsible of hospital water system contamination, as well as nosocomial infections. Besides, there are concerns that *L. anisa* could mask *L. pneumophila* water contamination [12]. Clinical manifestations described are mainly respiratory with eight reported pneumonia (seven immunocompromised (IC) patients) [8, 9] and 34 Pontiac fever during an outbreak in California [10]. Other manifestations included one pleural infection with probable pneumonia (IC) [5], one osteomyelitis secondary to pneumonia (IC) [4], one chronic endocarditis [6] and one mycotic aortic aneurysm [3] in both immunocompetent patients.

**Table 1:** Characteristics of the case reports of *Legionella anisa-*associated diseases, including the current case

Reference	Cases (n)	Sex	Age	Medical history	Significant IS	Presumed route of infection	Presentation	Diagnosis methods	Treatment
Tanabe et al. [3]	1	M	79	Y-graft replacement for an abdominal aortic aneurysm (3 years ago)	No	Unknown	Mycotic Aortic Aneurysm	PCR	LNZ, PFX 21d followed by LFX, CTM
Sanchez et al. [4]	1	M	51	Stage IV angioimmunoblastic T-cell lymphoma	Yes	Pneumonia (two months before)	Osteomyelitis of the patella	PCR, culture	MFX 56d
Bornstein et al. [5]	1	M	32	Lymph node carcinoma	Yes	Nosocomial pneumonia	Pleural infection	Culture	Deceased
Compain et al. [6]	1	F	58	Type 2 diabetes mellitus and grade II obesity	No	Unknown	Chronic endocarditis	PCR	LFX 21d
Thacker et al [7]	1	F	65	Type 2 diabetes mellitus	No	Pneumonia	Pneumonia	Culture	ERM
Vaccaro et al. [8]	1	F	36	–	No	Pneumonia	Pneumonia	PCR	LFX, CFX 10d
Head et al. [9]	6	3F, 3 M	31^a^	VIH ( tuberculosis or pneumocystosis co-infections)	Yes	Pneumonia	Pneumonia	PCR, culture	NA
Fenstersheib et al. [10]	34	NA	NA	NA	NA	Pneumonia	Pontiac fever	Serology	0
Current case	1	M	73	CLL	No	Direct inoculation	Arthritis	PCR	CPX 42d

*F* female, *M* male, *IS* immunosuppression, *LNZ* Linezolid, *PFX* Pazufloxacin, *LFX* Lefofloxacin, *CTM* Clarythromycin, *MFX* Moxifloxacin, *ERM* Erythromycin, *CFX* Cefixime, *CPX* Ciprofloxacin, *NA* not available

^a^Median

Immunologic response to *Legionella* infection is complex. *L. pneumophila* activates an important inflammatory response in hosts, with innate and adaptive responses. IFN-γ and TNFα are primarily responsible for immune clearance while CD4 + and CD8 + T cells additionally contribute to host defense [13]. Humoral response is considered feeble and does not provide prolonged immunity against the pathogen.

Arthritis caused by *Legionella* spp are rare, with only twelve cases previously described (Table 2). Seven were immunocompromised and two had kidney insufficiency (one moderate and one presumably non-severe given the arthritis antibiotic management). Median age at diagnosis was 71, range (51–90). Inoculation occurred most frequently through skin wound which are nonetheless rarely found at diagnosis. Some reports mentioned potential inoculation through corticosteroid injections [14–16]. However, acute arthritis following such injection could be unrecognized *legionella* infection potentiated by the induced local immunosuppression. Final, reactive arthritis has been a concern in one article and present with positive 16S RNA PCR with inflammatory fluid [17].

**Table 2:** Characteristics of the case reports of *Legionella* arthritis, including the current case

Reference	Sex	Age	Medical history	Significant IS	Joint (s)	Delay before diagnosis (days)	Strain	Diagnosis methods	Treatment
Dugar et al. [19]	M	56	RA, diabetes (CS, MTX)	Yes	L foot	2	*L. longbeachae*	Culture	AZM, MFX 42d
Just et al. [24]	F	71	Dermatomyositis (CS, MTX)	Yes	L knee		*L. bozemanii*	PCR, culture, serology	CPX 21d
Fernández-Cruz et al. [16]	F	83	RA (CS, MTX)	Yes	R knee	16	*L. micdadei*	PCR, culture	LFX, RFP 150d
Flendrie et al. [15]	F	58	SLE like disease (CS, MTX)	Yes	R knee		*L. dumoffii*	PCR, culture	CPX, RFP 90d
Huang et al. [21]	M	54	SLE (CS)	Yes	R MCP joints		*L. micdadei*	PCR, NGS, culture	LFX 60d
Ibranosyan et al. [20]	F	56	Anti-synthetase syndrome (CS, MTX, TCZ)	Yes	L wrist		*L. bozemanii*	PCR, culture	LFX, RFP 90d
Bemer et al. [22]	M	51	Thymoma (chemotherapy one year before)	Yes	R wrist and ankle, knees	30	*L. pneumophila S1*	UAT, culture, serology	OFX, RFP 21d
Naito et al. [18]	F	80	Kidney disease	No	Ankles	14	*L. pneumophila S1*	UAT, PCR	CPX
Thurneysen and Boggian [25]	M	70	Thymoma—hypogammaglobulinemia	No	R knee, L ankle		*L. pneumophila S1*	PCR, culture	CPX 90d
Linscott et al. [23]	F	80	None	No	R MCP joints	90	*L. pneumophila S4*	Culture, serology	Surgery
Banderet et al. [14]	F	90	Grade 3A kidney disease^a^	No	L wrist	21	*L. cincinnatiensis*	PCR, culture	AZM 21d
Current case	M	73	CLL	No	R wrist	42	*L. anisa*	PCR	CPX 42d

*F* female, *M* male, *L* left, *R* right, *CS* corticosteroids, *MTX* methotrexate, *TCZ* tocilizumab, *IS* immunosuppression, *UAT* urinary antigen test, *AZM* azithromycin, *CPX* ciprofloxacin, *LFX* levofloxacin, *RFP* rifampicin, *MFX* moxifloxacin

^a^According to KDIGO

The patients often presented few symptoms amid localized pain. Fever is rarely described (two cases with polyarthritis) [18, 19]. Delayed diagnosis is frequent with a median of 21 days, range (2–90). Polyarthritis seems to be a concern of L. pneumophila serogroup 1 (Lp1). Non-pneumophila strains are more frequently isolated in monoarthritis which is consistent with the direct mode of transmission [20]. Blood samples usually showed increase C-reactive protein, median 147 mg/L, range (< 5–254 mg/L). Fluid aspirate was hemorrhagic in two cases [20, 21], as our patient, with median neutrophil count of 80%, range (23–90).

Patients with significant immunosuppression (no isolated humoral deficiency as discussed previously) were older (median 80 vs 56 years) and had longer delayed diagnosis (median 32 vs 16 days).

Diagnosis was performed by 16S RNA PCR in each case except three. The other means of diagnosis were urinary antigen test for Lp1, serology, NGS and cultures. *Legionella* spp. require non-routine culture media for growth, especially BCYE. Successful cultures with chocolate agar and mycobacteria specific medium have been reported [22, 23]. Microbiologist must be aware of *Legionella* suspicion to perform such culture, which may lead to under-recognize diagnosis. Wide spreading of PCR might fill this gap. MALDI-TOF can be helpful for species identification [24].

There is no standard for antimicrobial therapy. Treatment consisted of fluoroquinolones in the majority of cases (9/11). Five patients had combination therapy (four rifampicin, one azithromycin). Data was missing in one patient. Median duration of antibiotic therapy for native septic arthritis was 42 days, range (21–90). One patient with knee prosthesis infection and was successfully treated with levofloxacin and rifampicin for five months. All strategies were effective.

We present the first case of septic arthritis caused by *L. anisa. Legionella* spp. should be suspected in arthritis, especially involving extremities and knee, with sterile standard culture, insidious evolution and compatible exposition. Concomitant pneumonia is uncommon but immunosuppression is not. Older age is probably a risk factor for *Legionella* arthritis.

## Data Availability

Not applicable.

## Abbreviations

NSAIDs: Non-steroidal anti-inflammatory drugs
RNA: Ribonucleic acid
PCR: Polymerase chain reaction
NGS: Next generation sequencing
BCYE: Buffered charcoal yeast extract
MALDI–TOF: Matrix assisted laser desorption ionisation/time of flight
F: Female
M: Male
LNZ: Linezolid
PFX: Pazufloxacin
LFX: Lefofloxacin
CTM: Clarythromycin
MFX: Moxifloxacin
ERM: Erythromycin
CFX: Cefixime
CPX: Ciprofloxacin
NA: Not available
L: Left
R: Right
CS: Corticosteroids
MTX: Methotrexate
TCZ: Tocilizumab
UAT: Urinary antigen test
AZM: Azithromycin
RFP: Rifampicin
